# A combinatorial approach for achieving CNS-selective RNAi

**DOI:** 10.1093/nar/gkae100

**Published:** 2024-02-13

**Authors:** Chantal M Ferguson, Bruno M D C Godinho, Dimas Echeverria, Matthew Hassler, Lorenc Vangjeli, Jacquelyn Sousa, Nicholas McHugh, Julia Alterman, Vignesh Hariharan, Pranathi Meda Krishnamurthy, Jonathan Watts, Eveny Rogaev, Anastasia Khvorova

**Affiliations:** RNA Therapeutics Institute, University of Massachusetts Medical School, Worcester, MA, 01605, USA; RNA Therapeutics Institute, University of Massachusetts Medical School, Worcester, MA, 01605, USA; RNA Therapeutics Institute, University of Massachusetts Medical School, Worcester, MA, 01605, USA; RNA Therapeutics Institute, University of Massachusetts Medical School, Worcester, MA, 01605, USA; RNA Therapeutics Institute, University of Massachusetts Medical School, Worcester, MA, 01605, USA; RNA Therapeutics Institute, University of Massachusetts Medical School, Worcester, MA, 01605, USA; RNA Therapeutics Institute, University of Massachusetts Medical School, Worcester, MA, 01605, USA; RNA Therapeutics Institute, University of Massachusetts Medical School, Worcester, MA, 01605, USA; RNA Therapeutics Institute, University of Massachusetts Medical School, Worcester, MA, 01605, USA; RNA Therapeutics Institute, University of Massachusetts Medical School, Worcester, MA, 01605, USA; RNA Therapeutics Institute, University of Massachusetts Medical School, Worcester, MA, 01605, USA; Department of Psychiatry, University of Massachusetts Medical School, Worcester, MA, 01605, USA; RNA Therapeutics Institute, University of Massachusetts Medical School, Worcester, MA, 01605, USA; Department of Medicine, University of Massachusetts Medical School, Worcester, MA, 01605, USA

## Abstract

RNA interference (RNAi) is an endogenous process that can be harnessed using chemically modified small interfering RNAs (siRNAs) to potently modulate gene expression in many tissues. The route of administration and chemical architecture are the primary drivers of oligonucleotide tissue distribution, including siRNAs. Independently of the nature and type, oligonucleotides are eliminated from the body through clearance tissues, where their unintended accumulation may result in undesired gene modulation. Divalent siRNAs (di-siRNAs) administered into the CSF induce robust gene silencing throughout the central nervous system (CNS). Upon clearance from the CSF, they are mainly filtered by the kidneys and liver, with the most functionally significant accumulation occurring in the liver. siRNA- and miRNA-induced silencing can be blocked through substrate inhibition using single-stranded, stabilized oligonucleotides called antagomirs or anti-siRNAs. Using APOE as a model target, we show that undesired di-siRNA-induced silencing in the liver can be mitigated through administration of liver targeting GalNAc-conjugated anti-siRNAs, without impacting CNS activity. Blocking unwanted hepatic APOE silencing achieves fully CNS-selective silencing, essential for potential clinical translation. While we focus on CNS/liver selectivity, coadministration of differentially targeting siRNA and anti-siRNAs can be adapted as a strategy to achieve tissue selectivity in different organ combinations.

## Graphical Abstract

**Graphical Abstract:**
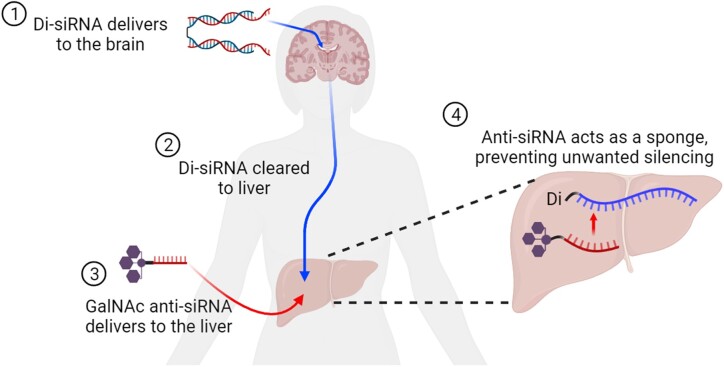
Graphical abstract created with Biorender.com

## Introduction

The advent of small interfering RNAs (siRNAs) provides an opportunity to silence gene expression in any organ at any desired time point. siRNAs prevent protein expression by targeting complementary mRNAs for degradation (1). For siRNAs, the base sequence determines the target mRNA, while the chemical architecture dictates pharmacokinetics and tissue delivery (2). For example, N-Acetylgalactosamine (GalNAc) conjugated siRNA architecture is an established, clinically validated platform for potent modulation of genes in the liver (3–5), with a single injection supporting 6–12 months of clinical efficacy (4). In the CNS, divalent (di)-siRNA scaffold is taken up by neurons and glial cells allowing for RNAi-mediated silencing in multiple CNS cell types (5). Upon intracerebroventricular (ICV) injection into mice, di-siRNAs travel in cerebrospinal fluid (CSF) to cause potent (>95%) and sustained (up to 6 months) target gene silencing throughout the CNS (5). For the di-siRNA, and many other chemically modified siRNAs, an asymmetric overhang increases efficacy, and the presence of a phosphorothioate backbone is essential for stability, tissue distribution, and enhanced tissue accumulation and efficacy (6,7). Although the inclusion of phosphorothioate modifications to the terminal linkages greatly enhances cellular uptake, phosphorothioate-enhanced cellular uptake is not receptor mediated or cell type specific and thus, can result in accumulation in cell types outside of the CNS or target organ. Di-siRNAs are partially cleared from the CSF into the systemic circulation and are subsequently cleared from systemic circulation mainly via the liver (8,9). At high doses, systemic clearance may result in cellular accumulation and detectable silencing in clearance tissues that express the target mRNA.

While for most targets, unintended modulation of expression in the liver is not problematic, for some, achieving complete tissue selectivity is essential. One of these targets is Apolipoprotein E (APOE), where CNS and liver APOE are spatially and functional distinct. The APOE4 allele remains the strongest genetic risk factor for developing late onset Alzheimer’s disease (AD) and is a compelling target for modulation in AD. The majority of APOE is produced in the (i) central nervous system, where it is primarily expressed by astrocytes (and to a lesser extent, neurons) to transport lipids between cells (10) and modulate the inflammatory response (11), and (ii) the liver, where it is secreted by hepatocytes to facilitate lipid uptake into peripheral tissues via low-density lipoprotein (LDL) receptors (12,13). There are three allelic variants of APOE: APOE2, APOE3 and APOE4. While APOE4 increases the risk of developing AD and decreases the age of clinical onset, the presence of APOE2 confers protection against AD. Conversely, individuals carrying APOE2 have a higher risk of developing atherosclerosis due to the loss of APOE’s key function in cholesterol processing. Genetic removal of APOE (mouse and human) in AD models reduces Aβ plaques and improves cognitive outcomes (14–18). However, peripheral APOE2 expression and loss of liver Apoe in mice cause severe atherosclerosis (19). Thus, unwanted silencing of APOE in the liver may have functional impacts, and strategies to ensure organ specific APOE silencing are necessary.

In summary, APOE is highly expressed in both organs, has distinct spatial and functional characteristics in both organs, and is a relevant target for many neurodegenerative, liver and cardiac diseases. Thus, we chose APOE as a model target to demonstrate the feasibility of achieving fully selective CNS RNAi.

Chemical modifications and the inclusion of bioconjugates direct siRNA delivery and silencing to specific organs and cell types of interest. However, siRNAs are typically cleared from the body by the liver or/and kidneys where their unintended accumulation can direct non-specific target gene modulation. Thus, preventing unwanted silencing in clearance tissues is necessary to achieve fully tissue-selective effects. siRNA silencing depends on efficient interaction between the siRNA guide strand and the RNA-induced silencing complex (RISC). This siRNA–RISC interaction can be sterically blocked by the introduction of fully modified, single-stranded Antisense Oligonucleotides (ASOs) that are complementary to the siRNA guide strand sequence (20,21). The Stoffel lab, in collaboration with Alnylam, was the first to describe the short ASO’s ability to inhibit loaded RISC complex in 2005 and named the compounds ‘AntagomiRs’ (21). The naming was defined by the functional ability to inhibit miRNA activity. The same configuration was successfully used to inhibit the activity of synthetic siRNAs, reversing the siRNA activity, and called ‘ReversiRs’ (20). The terms antagomir, reversir and anti-siRNA all describe ASOs that are complementary to the loaded RISC and represent a biochemically identical phenomenon. The names are derived from the functional application rather than the biological mechanism – when anti-siRNAs are used for miRNA inhibition, they are termed antagomirs (21); when used for reversing of RNAi-induced silencing, they are called reversiRs (20). Here, we use the term anti-siRNA to ensure clarity that the ASOs are blocking non-desired activity, rather than reversing the intended function.

Here, we explore whether combinatorial administration of siRNAs and ‘anti-siRNAs’ in differentially targeted chemical scaffolds could be used to achieve tissue selectivity. In two therapeutic paradigms, we show that GalNAc-conjugated anti-siRNAs rescue and block off-tissue silencing of *APOE* in the liver without any impact on CNS activity.

## Materials and methods

### Reagents

Anti-ApoE antibody (Abcam, 183597); 1:200 dilution

Anti-Vinculin antibody (Invitrogen, 700062); 1:1000 dilution

Abcam LDL and HDL cholesterol quantification kit (ab65390)

RNAlater (Invitrogen #AM7020)

Quantigene 2.0 homogenizing buffer (Invitrogen, QG0517)

Proteinase K (Invitrogen, 25530–049)

QuantiGene probesets: mouse HPRT (SB-15463), mouse PPIB (SB-10002), mouse apoE (SB-13611), human apoE (SA-10292), human PPIB (SA-10003)

WES by ProteinSimple, 16–230 kDa plate (SM-W004)

Human APOE ELISA (ab108813)

Eagle’s Minimum Essential Media (EMEM) (ATCC 302003), Fetal Bovine Serum (FBS) (Corning 35010CV)

### Biological resources

HepG2 cell line (ATCC HB-8065)

FBV/NJ mice (001800-JAX)

Humanized APOE4 mice (027894-JAX) (breeding pairs)

### Animal studies

All experimental studies involving animals were approved by the University of Massachusetts Medical School Institutional Animal Care and Use Committee (IACUC Protocols #A-2411 and #A-1744) and performed according to the guidelines and regulations therein described. Wild-type adult (6–8 weeks) female FBV/NJ mice (001800-JAX) were obtained from Jackson laboratory. Humanized APOE4 (homozygous) (027894-JAX) mice (breeding pairs) were obtained from Jackson laboratory and bred in house.

### Statistical analysis

Each *n* represents an independent biological sample. All graphs show means ± S.D. All statistics were performed using Prism GraphPad v.8, using either two-tailed, unpaired *t*-tests or one-way ANOVA with Dunnett’s correction. Box plots: center line, median; top of box, 75% quartile; bottom of box, 25% quartile; whiskers, minimum and maximum.

### Custom divalent support synthesis

Divalent custom solid support synthesis was previously reported (5). The method is described briefly below.


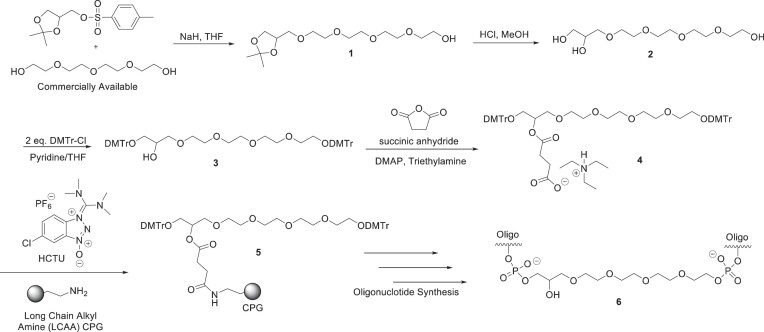


Excess tetraethylene glycol was treated with sodium hydride in tetrahydrofura (THF) at 0°C, and Solketal tosylate dissolved in THF was added dropwise to produce the monofunctionalized glycol (1). The acetal was removed by treatment with HCl in methanol, and both primary hydroxyl groups were protected with the dimethoxytrityl protecting group to produce compound (3). Succinic anhydride in the presence of triethylamine (TEA) was used to produce the succinate (22), which was subsequently used to functionalize native long chain alkyl amine controlled pore glass (CPG, 1000 or 500A) or polystyrene type resins using traditional peptide coupling reagents. These functionalized resins were then used to synthesize the sense strands for divalent siRNA using standard oligonucleotide synthesis techniques.

### Custom GalNAc solid support synthesis

The synthesis of the custom GalNAc support was previously reported (23). The method is described briefly below.


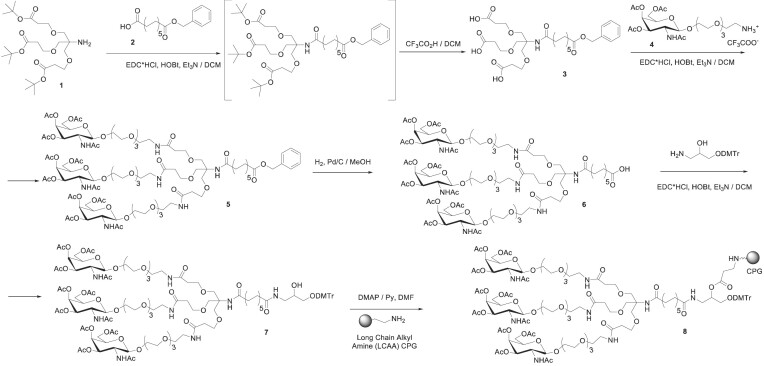


The amino-polyether tritert-butyl ester (1) coupled with 12-(benzyloxy)-12-oxododecanoic acid. The crude mixture was extracted and isolated without purification. The tbutyl ester was removed by treatment with 25% Triflouroacetic acid (TFA) solution in dichloromethane (DCM). The resulting triacid was then coupled with the peracetylated GalNAc functionalized at the anomeric position with a triethylene glycol aminolinker (22) resulting in compound (24) in roughly 75% yield. The benzoyl protecting group was removed by H_2_/Pd/C in methanol. The resulting carboxylic acid (4) was coupled with a dimethoxy trityl protected 3-amino-1,2-propanediol producing compound (5). The secondary hydroxyl was then reacted with succinic anhydride and coupled with long chain alkyl amine CPG using standard peptide coupling reagents. The resulting CPG was used to synthesize any 3′-GalNAc containing oligonucleotides/anti-siRNAs using standard oligonucleotide synthesis techniques.

### Vinyl phosphonate deprotection

The vinyl phosphonate (VP)-containing oligonucleotides, still on solid support, were treated post synthesis with an anhydrous mixture of trimethylsilyl bromide/acetonitrile (ACN)/dimethylformamide (DMF)/pyridine (3:9:9:1) for 1 h at room temperature with gentle agitation. The reaction was then quenched with water and the CPG was then rinsed with ACN, DCM and allowed to dry, before being deprotected normally as described below.

### Standard oligonucleotide synthesis

Oligonucleotides were synthesized using modified (2'-F, 2'-*o*-Me, locked nucleic acid [LNA]) phosphoramidites with standard protecting groups. Phosphoramidite solid-phase synthesis was done on a MerMade12 (BioAutomation) using modified protocols. Unconjugated oligonucleotides were synthesized on CPG functionalized with a long-chain alkyl amine (LCAA) and unylinker terminus (Chemgenes). Cholesterol CPG was purchased from Chemgenes (Wilmington MA) Product # N-9166–05. GalNAc-conjugated oligonucleotides were grown on custom 3′-GalNAc-CPG (23); divalent oligonucleotides (DIOs) were synthesized on modified solid support (5), and VP phosphoramidite was synthesized as described. Phosphoramidites were prepared at 0.1 M in anhydrous ACN, with added dry 15% DMF in the 2′-OMe U amidite. 5-(Benzylthio)-1H-tetrazole (BTT) was used as the activator at 0.25 M. Detritylations were performed using 3% trichloroacetic acid in DCM. Capping was done with non-tetrahydrofuran-containing reagents CAP A, 20% n-methylimidazole in ACN and CAP B, 20% acetic anhydride (Ac_2_O) and 30% 2,6-lutidine in ACN (synthesis reagents were purchased at AIC). Sulfurization was performed with 0.1 M solution of 3-[(dimethylaminomethylene)amino]-3H-1,2,4-dithiazole-5-thione (DDTT) in pyridine (ChemGenes) for 3 min. Phosphoramidite coupling times were 3 min for all amidites used.

### Deprotection and purification of oligonucleotides

Divalent and conjugated oligonucleotides (DIO, GalNAc) were cleaved and deprotected with standard conditions using aqueous ammonia at 55°C for 16 h. VP-containing oligonucleotides were cleaved and deprotected as described in O’Shea et al., 2018 (25). Briefly, CPG-containing VP-oligonucleotides were treated with a solution of 3% diethylamine (DEA) in aqueous ammonia at 35°C for 20 h. Solutions containing deprotected oligonucleotides were filtered to remove the CPG and dried under vacuum in Speed-vac. The resulting pellets were re-suspended in 5% ACN in water. Purification was performed on an Agilent 1290 Infinity II HPLC system, equipped with a Source 15Q anion exchange column (GE Healthcare) using the following conditions: eluent A, 20% ACN, 20 mM sodium acetate, pH 7; eluent B, 1 M sodium perchlorate in 20% ACN; gradient, 10% B (3 min) to 35% B (18 min) at 60 °C. Peaks were monitored at 260 nm. Pure fractions were collected and dried in Speed-vac. Oligonucleotides were re-suspended in 5% ACN and desalted through fine Sephadex G-25 media (GE Healthcare) and lyophilized.

### LC–MS analysis of oligonucleotides

The identity of oligonucleotides was verified by LC–MS analysis on an Agilent 6530 accurate mass Quadruple Time of Fight (Q-TOF) using the following conditions: buffer A, 100 mM hexafluoroisopropanol (HFIP)/9 mM TEA in LC–MS grade water; buffer B, 100 mM HFIP/9 mM TEA in LC–MS grade methanol; column, Agilent AdvanceBio oligonucleotides C18; gradient 0% B (1 min), 0–40% B (8 min), temperature: 45°C; flow rate, 0.5 ml/min. LC peaks were monitored on UV (260 nm). MS parameters: source, electrospray ionization; ion polarity, negative mode; range: 100–3200 *m*/*z*; scan rate: 2 spectra/s; capillary voltage: 4000; fragmentor: 180 V. Reagents were purchased from Fisher Scientific, Sigma Aldrich and Oakwood Chemicals, and used as per manufacturer’s instructions, unless otherwise stated.

### Screening and dose–response studies

HepG2 cells were cultured in EMEM 10% FBS and grown to confluency in T75 flasks. For screening experiments, siRNAs were diluted to a concentration of 1.5 μM in 50 μl of Opti-MEM. Diluted siRNAs (50 μl) were added to a 96-well plate using a multichannel pipette. Next, HepG2 cells were added to the same 96-well plate at a concentration of 25 000 cells per well in 50 μl of EMEM media with 6% FBS (final concentration 3%). Each siRNA was tested in triplicate. Cells were incubated at 37°C for 72 h at which point mRNA concentration was measured using QuantiGene, which was performed according to the manufacturer’s instructions.

### Stereotactic ICV injections

Approximately 10 μl of di-siRNA was administered bilaterally (5 μs per ventricle) into the lateral ventricles of mice as previously described (5). Briefly, mice were anesthetized using Avertin and prepared using standard aseptic technique. Stereotaxic devices were using to hold injection needles and identify injection location. After the identification of the bregma, the needle was placed 1 mm laterally, 0.2 mm posterior and 2.5 mm caudally. Injection was performed at 500 nl/min. Mice were then monitored until fully sternal.

### Subcutaneous injections

GalNAc-conjugated siRNAs were injected subcutaneously (SC) into mice. Each animal received a 10 mg/kg dose in 200 μl of PBS. For anti-siRNAs, mice received 1 mg/kg SC in 200 μl of PBS.

### mRNA quantification

mRNA quantification was performed as described (5). Briefly, tissue punches were stored in RNAlater (Invitrogen #AM7020) and homogenized in Quantigene 2.0 homogenizing buffer (Invitrogen, QG0517) with proteinase K (Invitrogen, 25530–049). mRNA was detected according to the Quantigene 2.0 protocol using the following probe sets: mouse HPRT (SB-15463), mouse PPIB (SB-10002), mouse apoE (SB-13611), human apoE (SA-10292) and human PPIB (SA-10003).

### APOE protein quantification

For analysis of APOE protein expression in mouse brain samples, WES by ProteinSimple was used as previously described in (5) and (26). Briefly, tissue punches were collected as above and flash-frozen and placed at −80°C. After addition of radioimmunoprecipitation assay buffer (RIPA) buffer with protease inhibitors, samples were homogenized and stored at −80°C. Protein amount was determined using Bradford Assay. Samples were diluted in 0.1× sample buffer (ProteinSimple) to ∼0.2–0.4 μg/μl. Anti-APOE antibody (Abcam, 183597) was diluted 1:200 in antibody diluent (ProteinSimple), and loading control, anti-vinculin (Invitrogen, 700062), was diluted 1:1000 in antibody diluent. The assay was performed as described by ProteinSimple protocol using the 16–230 kDa plate (SM-W004). The separation electrophoresis and immunodetection are performed automatically in the capillary system using the default system settings. Once loaded, the separation electrophoresis was performed automatically. Results were analyzed using the Compass for Protein Simple software.

### Cholesterol

Serum cholesterol was measured using the Abcam low density lipoprotein (LDL) and high density lipoprotein (HDL) cholesterol quantification kit (ab65390). LDL and HDL were separated using the included precipitation buffer that uses a water soluble non-ionic polymer to precipitate the fractions (27). The assay uses cholesterol esterase to hydrolyze cholesteryl ester into free cholesterol. Next, cholesterol oxidase acts on free cholesterol and to produce a color at 570 nm that is proportional to the amount of cholesterol in the sample. Briefly, serum was collected prior to euthanasia. 2× buffer was added to 50 μl of serum and incubated at room temperature for 10 min. Samples were spun for 10 min at 2000 rpm, and the supernatant (LDL fraction) was placed in a separate tube. The pellet (HDL fraction) was resuspended in 200 μl PBS. The samples were diluted, and cholesterol levels analyzed according to the package instructions.

### APOE plasma quantification

Levels of secreted APOE protein (largely produced by hepatocytes) in plasma samples were evaluated pre-injection, and 24-, 48-, 72-, 96-h, 5-, 6-, 8-, 10- and 12-day post injection using ELISA (ab108813).

## Results

### Selection and synthesis of oligonucleotides

**Figure 1.**
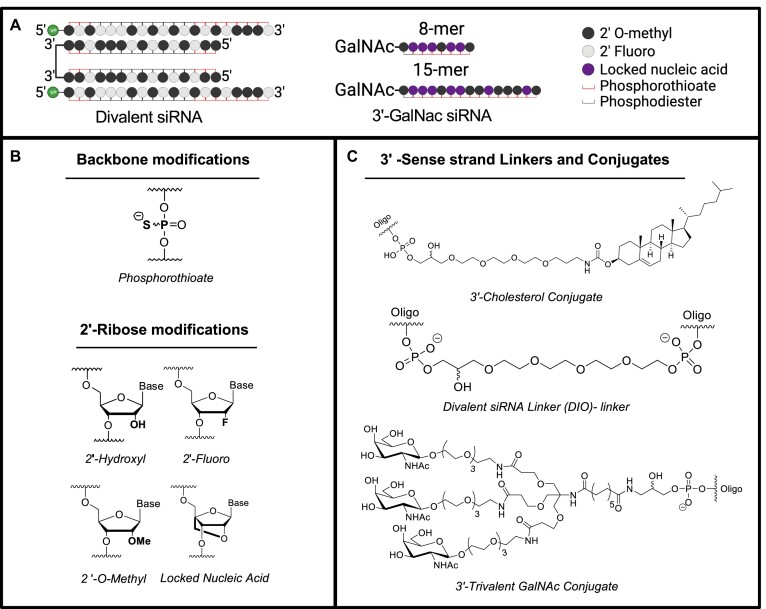
Chemical structure of nucleoside modifications and 3′-linkers and conjugates. (**A**) Schematic representing the structure and chemical modifications of di-siRNAs (left) and GalNAc anti-siRNAs (middle). (**B**) Chemical structure of oligonucleotide backbone phosphorothioate modification and 2′-OH modifications, including the hative 2′-OH, 2′-fluoro, 2′-*o*-methyl and LNA. (**C**) Chemical structure of cholesterol conjugate (top), divalent siRNA linker (middle) and trivalent GalNAc conjugate (bottom).

For the experiments presented here, we utilized chemically modified oligonucleotides and relied upon three previously identified conjugates for specific purposes (Figure 1). Full chemical stabilization is essential for conjugate-mediated delivery *in vivo*, where siRNAs must survive for extended periods in endosomes/lysosomes compartment to support sustained target gene modulation. The inclusion of fluoro and *o*-methyl modifications to the native RNA 2′-hydroxyl increases stability and efficacy both *in vitro* and *in vivo* (Figure 1B) (28,29).

We, and others, continue to evaluate the impact of changing the degree of modification, the balance of modifications within an siRNA and the positional effects of chemical modification on siRNA efficacy and distribution. For these studies, we chose a balanced pattern, with an approximately equal number of *o*-methyl and fluoro modifications on each siRNA strand. Here, we included a run of fluoro modifications on the antisense strand around the seed-binding site to enhance seed site affinity for both *in vitro* and *in vivo* siRNAs (Figure 1A,B). Locked nucleic acid (LNA) modifications, in which a carbon bridge between the 2′-oxygen and the 4′-carbon position (Figure 1B), increase the stability and affinity of oligonucleotides to their complementary mRNA sites. Here, we used LNA modifications in the anti-siRNA oligonucleotides to enhance affinity to the APOE-siRNA antisense strand and describe the rationale for the position of LNA modifications below.

We used three chemical conjugates in these studies: cholesterol conjugation to deliver siRNAs into cells for *in vitro* screening experiments, divalent conjugation for siRNA delivery throughout the CNS and GalNAc conjugation for delivery to the liver. The conjugation of hydrophobic entities, like cholesterol (Figure 1C) (Cholesterol CPG: Chemgenes #N-9166–05), to fully chemically stabilized siRNA, allows for efficient and passive internalization and productive gene modulation in virtually all cell types *in vitro* without the use of transfection reagents (30). Cellular uptake of oligonucleotides without transfection reagents is called gymnotic delivery, and, when directly translated from the original Greek, means naked ‘gymnos’ delivery (30). The use of gymnotic delivery for *in vitro* screening simplifies the workflow and provides a better experimental design predicting compound *in vivo* behavior.

The divalent siRNA linker (Figure 1C), first published in Alterman *et al.*, 2019 (5), contains a triethylene glycol linker connecting the two sense strands together at the 3′-terminus, connecting the two functional siRNAs (Figure 1C). The divalent linker was used to efficiently deliver siRNAs throughout the CNS and was conjugated to siRNAs targeting *APOE* to silence expression throughout the CNS. The trivalent GalNAc conjugate (Figure 1C) was synthesized in house (Materials and methods) and used to direct delivery of anti-siRNAs to hepatocytes in the liver, the main site of systemic *APOE* expression. The expected and observed mass (g/mol) of each oligonucleotide strand used for *in vivo* experiments and the extinction coefficient are shown in Supplementary Table S2.

### Identification of siRNAs targeting *APOE*

We designed a panel of *Apoe*-targeting siRNA candidate sequences for mouse *Apoe* and human *APOE* using an internally validated algorithm with methods based on the principles described in Birmingham *et al.* (31). Ideal candidate sequences have a GC content of <45%, do not contain toxic motifs such as G-quadraplexes and span the length of the mRNA sequence.

We performed initial screens using cholesterol-conjugated siRNAs at a 1.5 μM dose to identify hit sequences and validated the hits in eight-point dose–response studies by serially diluting from the top dose of 1.5 μM. We previously published screens and validations identifying potent sequences targeting mouse *Apoe* and chose APOE-1134 as the lead mouse-targeting sequence (32). Here, using cholesterol conjugated siRNAs, we screened 12 siRNA sequences targeting human *APOE* in human HepG2 cells and identified two hit sequences (Supplementary Figure S1a). Hits were validated in seven-point dose–response studies and identified APOE-1142 as the most potent and efficacious sequence (IC50: 298.2 nM) (Supplementary Figure S1b).

### siRNAs in CNS- and liver-targeting configurations enable tissue-selective *apoe* silencing in wild-type mice

**Figure 2.**
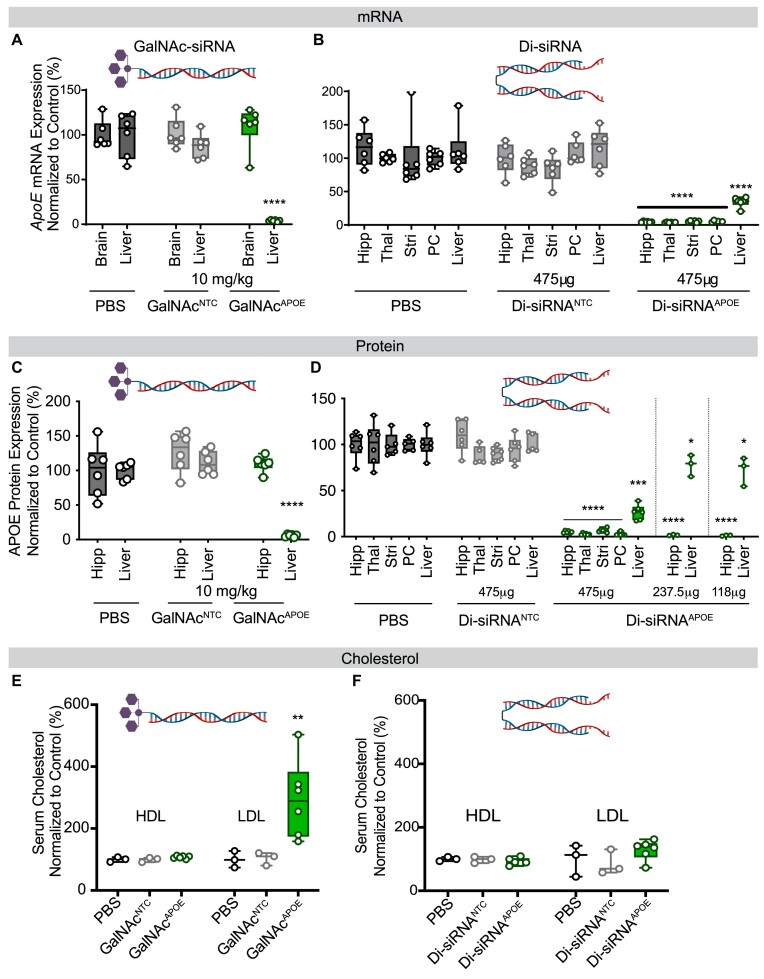
GalNAc and divalent siRNA chemical scaffolds support tissue specific *Apoe* silencing. (**A,B**) *Apoe* mRNA expression in the brain and liver after administration of (A) GalNAc^APOE^ (SC) or (B) di-siRNA^APOE^ (ICV). (**C,D**) protein in the brain and liver after administration of (C) GalNAc^APOE^ or (D) di-siRNA^APOE^. For di-siRNA^APOE^ (ICV) three doses were tested: 475, 237 and 118 μg, showing dose-dependent recovery of silencing effects in the liver. (**E**,**F**) Serum cholesterol (HDL and LDL) after silencing (E) liver or (F) CNS *Apoe* (high dose: 475 μg). Mice: adult female FBV/NJ (8 weeks); Timepoint: 1 month post-administration; Dose: GalNAc: 10 mg/kg, *n* = 6/group, di-siRNA: 475 (*n* = 6/group), 237 (*n* = 3) and 118 μg (*n* = 3). Statistical analysis: one-way ANOVA using Prism. Error bars are SD (****P< 0.0001, ***P< 0.002, **P< 0.01, *P< 0.05).

To explore the feasibility of silencing different pools of Apoe, we engineered the siRNAs targeting mouse *Apoe* in either a liver-active (GalNAc^APOE^) (3–5) or CNS-active (di-siRNA^APOE^) (5) chemical configuration (Figure 2; Supplementary Tables S1, S2 for sequence information).

In wild-type mice, one-month post-injection, GalNAc^APOE^ resulted in potent *Apoe* mRNA and protein silencing in the liver (>95%, *P*< 0.0001) compared to PBS and non-targeting control (NTC) groups (10 mg/kg SC; *n* = 6/group). We observed no detectable silencing of *Apoe* mRNA or protein in brain samples, as expected since systemically administrated oligonucleotides do not cross the blood–brain barrier. Administration of di-siRNA^APOE^ into the lateral ventricles (ICV) also resulted in potent *Apoe* mRNA and protein silencing in the brain (>95%, *P*< 0.0001) compared to both PBS and NTC groups (475 μg ICV; *n* = 6/group). A portion of the siRNA dose administered into the brain escapes the CNS and is cleared from circulation via the liver (33–35). Thus, di-siRNA administered into the brain also resulted in reduction of liver *Apoe* mRNA and protein (∼75%, *P*< 0.0001). To mitigate off-tissue silencing, we reduced the dose of di-siRNA 2- and 4-fold. Lower doses (237 or 118 μg) of di-siRNA^APOE^ achieved similar brain *Apoe* silencing (>95%, *P*< 0.0001) while reducing the degree of silencing in the liver (∼20%, *P*< 0.05) (Figure 2D, Supplementary Figure S2b,c). We observed no changes in blood chemistry markers or complete blood counts at 2- and 21-day post-treatment of either siRNA, indicating lack of liver/kidney toxicity (Supplementary Table S3).

As expected, complete liver Apoe silencing by GalNAc^APOE^ significantly increased serum LDL levels (>300%, *P*< 0.01) (Figure 2E), whereas di-siRNA^APOE^-mediated brain Apoe silencing (which also reduced liver *ApoE* by ∼75%), had no effect on serum LDL one-month post-injection (Figure 2E). Collectively, these data provide further evidence that two spatially distinct pools of *Apoe* exist in wild-type mice. In the short-term, Apoe does not exit the CNS to replenish systemic Apoe or alter systemic cholesterol homeostasis, and systemic Apoe does not rescue the loss of brain Apoe.

### Chemical engineering to achieve selective CNS target silencing

Although in the short-term, partial reduction of liver *Apoe* by di-siRNA does not translate into detectable serum cholesterol changes in wild-type or AD mouse models (36) and can be controlled by dose reduction, long-term partial reduction may have a more significant impact. Thus, we sought to devise a method to block any silencing of *APOE* in the liver.

**Figure 3.**
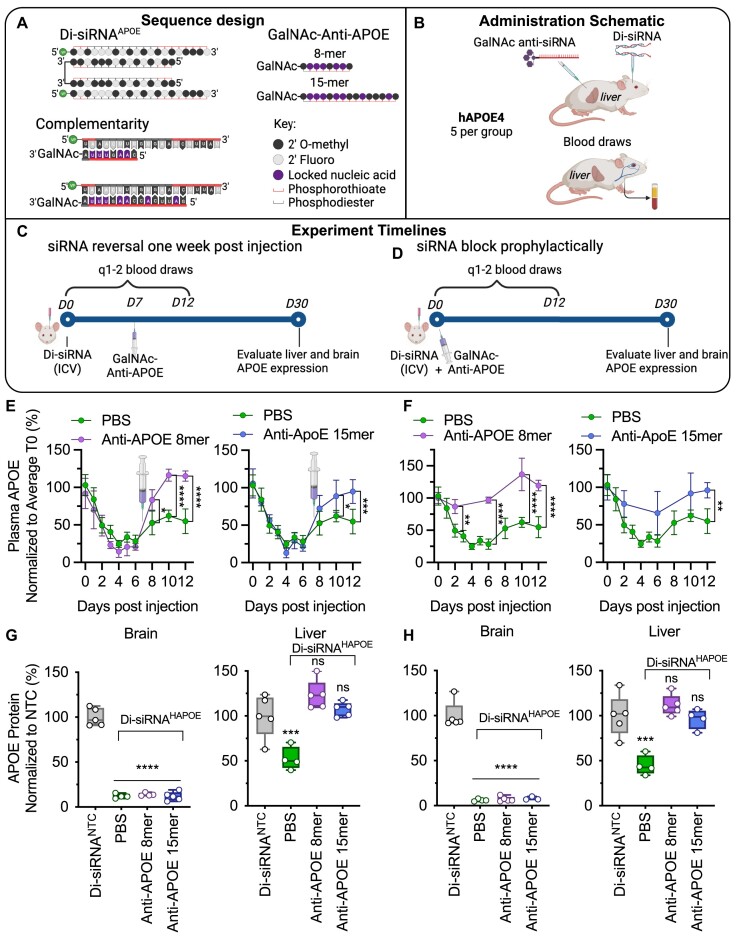
GalNAc-conjugated ASOs efficiently block and reverse di-siRNA-induced liver *APOE* modulation. (**A**) Di-siRNA and GalNAc-conjugated anti-siRNA designs. (**B**) Administration schematic into humanized APOE4 mice showing ICV administration of di-siRNA, subcutaneous (SC) administration of GalNac-anti-siRNAs, and blood draws from the facial vein to measure systemic APOE levels. (**C,D**) Experimental timeline for staggered (reversal) and co-administration (prophylactic block) of di-siRNA and anti-siRNA. (**E**) Loss and rescue of plasma (hepatic) APOE after ICV administration of di-siRNA^HAPOE^ followed by SC administration of GalNAc-anti-siRNA 7 days later (compared to PBS). Purple (left) shows anti-APOE-8mer and blue (right) shows anti-APOE-15mer. (**F**) No loss of plasma APOE (compared to PBS) after concurrent administration of GalNAc-anti-siRNA and di-siRNA^HAPOE^. Left panel (purple) shows anti-APOE-8mer and right panel (blue) shows anti-APOE-15mer. (**G,H**) Sustained silencing of APOE protein in the hippocampus (left) and rescue of liver APOE protein expression (right) after (G) delayed or (H) concurrent administration of GalNAc-anti-APOE. Purple shows anti-APOE-8mer and blue shows anti-APOE-15mer. Dose: Di-siRNA: ICV, 475 ug; Anti-APOE: SC, 1 mg/kg. *n* = 4–5/group plasma APOE measured with human APOE ELISA; Tissue protein measured using the WES western blot system. Statistical analysis: one-way ANOVA using GraphPad Prism. Error bars are SD (****P< 0.0001, *** *P*< 0.002, ** *P*< 0.01, * *P*< 0.05). Schematic A-D created with Biorender.com

For these experiments, we moved to targeting human *APOE* in the humanized APOE4 mouse model as it has greater translatability and used the APOE-1142 sequence identified in Supplementary Figure S1. siRNA silencing can be sterically blocked by fully modified single-stranded oligonucleotides that are complementary to the siRNA sequence (20,21). We optimized this technology and explored whether combinatorial administration of siRNA (di-siRNA via ICV in CNS) and anti-siRNA (GalNac-anti-siRNA SC) by different routes and using different chemical scaffolds could block any unwanted silencing in the liver. Specifically, we designed and synthesized two anti-siRNA variants (8- and 15-mer), whose sequences were complementary to the di-siRNA^HAPOE^ guide strand (Figure 3A) (20,37). Prior characterization of REVERSIR showed greatest efficacy with oligo lengths of 8–9 mer, moderate activity when length was increased to 15- and 21-mer, and inactivity when length was decreased to 7-mer (20). Thus, we chose lengths of 8- and 15-mer to evaluate varying efficacy in blocking unwanted silencing.

Several locked nucleic acid modifications were introduced at positions 2, 3, 4, 6, 7 and 14 (15-mer) from the 3′-end to enhance affinity with target siRNAs and to enhance metabolic stability (Figure 3A and Supplementary Table S1). The specific LNA positions were chosen to enhance binding initiation, enhance affinity to the seed region, and based on results showing the importance of including an LNA at position 6 (20). ‘Anti-siRNAs’ were then conjugated to GalNAc with a cleavable linker to drive preferential accumulation in liver upon systemic administration (20). The addition of a cleavable linker between GalNAc and anti-siRNA has previously been shown to enhance the efficacy of anti-RISC antisense compounds and conjugated siRNAs (7,20). There are different variants of cleavable linkers, and their stability needs to be optimized to be stable in serum during the distribution phase but cleavable intracellularly in the endosomes/lysosomes. The introduction of a dT phosphodiester linker provides the necessary properties and is methodically simple. Thus, the dT-PO cleavable linker was used to connect GalNAc to anti-siRNAs.

To evaluate the ability of anti-siRNAs to selectively reverse and block di-siRNA^HAPOE^ silencing in the liver, we administered high dose di-siRNA (475 μg) via ICV injection to humanized *APOE4* mice (JAX#027894). Seven days later, we administered 1 mg/kg GalNAc-anti-siRNAs subcutaneously (either 8- or 15-mer) (Figure 3B,C). We evaluated APOE plasma protein levels over time as a read out for *APOE* silencing in the liver. Forty-eight hours after di-siRNA^HAPOE^ injection, plasma APOE levels dropped to ∼30% of initial levels (Figure 3E). Within 24 h after administration of anti-siRNA (either 8- or 15-mer), plasma APOE levels began to recover and within 3 days, APOE expression returned to pre-di-siRNA treatment levels (Figure 3E). Consistent with the rescue of plasma APOE, animals treated with anti-siRNAs post-administration of di-siRNA showed no reduction in liver APOE protein relative to controls one-month post injection (Figure 3G and Supplementary Figure S3 for western blots). As GalNAc anti-siRNAs do not cross the blood–brain barrier, SC administration had no effect on brain APOE silencing (>95%, *P*< 0.0001) (Figure 3G). Therefore, tissue selective reversal of RNAi activity in the liver enables silencing of *APOE* selectively in the CNS.

In a separate experiment, we co-administered GalNac-anti-siRNAs and di-siRNA^HAPOE^ to determine if unintended silencing in the liver could be completely blocked (Figure 3D). Co-administration of anti-siRNAs (both 8- and 15-mer) resulted in no reduction in plasma APOE, suggesting that di-siRNA silencing in liver was completely blocked by liver-targeting anti-siRNAs (Figure 3F). Consistent with the reversal paradigm, anti-siRNAs completely blocked reduction in liver APOE protein relative to controls one-month post injection (Figure 3H and Supplementary Figure S2 for western blots) and had no impact on brain APOE protein levels (Figure 3H).

In both experiments, SC injection of PBS control showed no rescue of di-siRNA-induced liver APOE modulation (Figure 3G [right] and Figure 3h [right]), confirming the effect is due to the anti-siRNAs. Due to the need to limit the number of animals used in the study, a PBS control, but not a random GalNAc anti-siRNA control, was used. The chances that the observed reduction in APOE modulation in the liver was due to non-specific effects of GalNAc anti-siRNA administration are low, as there are extensive public data on the specificity and use of GalNAc-modified ASOs in the liver. Analysis of liver toxicity 1-month post injection showed no significant differences between groups (Supplementary Table S4). While both anti-siRNA structures prevented silencing of *APOE* in the liver across experiments, the shorter anti-siRNA (8 mer) was more effective than the longer compound (15 mer). This finding is consistent with previous data showing an impact of anti-siRNA design on efficacy (37). Taken together, these data demonstration that anti-siRNAs block unwanted RNAi activity in the liver, enabling tissue selective silencing of *APOE* in the CNS with divalent siRNAs.

**Figure 4.**
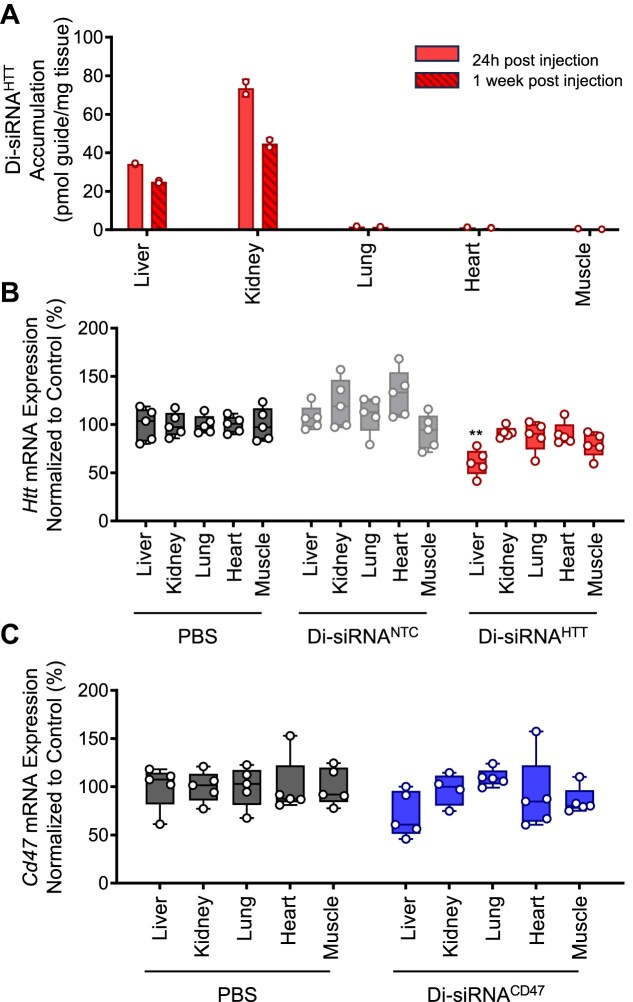
Divalent siRNA systemic distribution is primarily limited to the liver and kidney. Di-siRNAs targeting uniformly expressed genes *HTT* and *CD47* were administered to mice (20 mg/kg, ∼437 μg). (**A**) Systemic accumulation of *HTT* targeting di-siRNA at 24 h and 1-week post-administration (measured with peptide nucleic acid (PNA) hybridization assay) showing that systemic exposure is limited to two major clearance tissues: liver and kidney. *Htt* (**B**) and *Cd47* (**C**) mRNA levels 2-week post-administration (QuantiGene) showing statistically significant silencing only with di-siRNA targeting HTT in the liver. N = 6/group. Statistics: error bars STDEV, one-way ANOVA with Tucker correction for multiple comparisons; ** *P*< 0.01.

To confirm that blocking di-siRNA activity in the liver would mitigate the off-tissue effects of di-siRNAs, we evaluated the level of systemic tissue accumulation and silencing for two additional di-siRNAs targeting Huntingtin *(Htt)* and *Cd47* mRNA (Figure 4). Twenty-four hours and one-week post-administration, we observed that systemic exposure was limited to the liver and kidney (Figure 4A). We also evaluated mRNA silencing at 2-week post-administration and observed significant mRNA silencing only in the liver of animals treated with di-siRNA targeting *Htt* (Figure 4B) and no silencing when targeting Cd47 (Figure 4C). Based on these results, blocking the off-tissue effect of di-siRNAs in the liver is sufficient to achieve CNS-selective silencing.

## Discussion

Tissue-selective RNAi is a holy grail of oligonucleotide therapeutics. GalNAc conjugated oligonucleotides promote tissue selectivity via receptor mediated uptake in the liver, which is one of the primary clearance organs. For extrahepatic delivery, some modulation of expression in clearance tissues, in addition to the target organ, is expected and difficult to avoid. Unwanted silencing in clearance tissues can be avoided by reducing the administered dose. However, as higher doses translate to longer duration of effect, lower doses may result in reduced clinical efficacy. Here, we describe a new technological twist for achieving tissue selectivity in the CNS. We use a combination of different routes of administration (ICV and SC) and different scaffolds (divalent and GalNAc) to promote orthogonal siRNA distribution and achieve tissue selectivity. The approach presented here uses similar technology to antagomirs and reversirs. However, the main conceptual idea builds upon prior studies by using different delivery scaffolds from the primary oligonucleotide.

In these experiments, we used *APOE* as a model target gene. Like other genes, *APOE* is expressed by different cell types in multiple organ systems with organ-specific functions and effects. It’s role in systemic lipid homeostasis is indispensable; however, presence of the *APOE4* allele is the most significant genetic risk factor for developing late onset AD and is implicated in many other neurodegenerative conditions. Thus, identifying strategies to reduce or block *APOE* expression in the CNS without impacting hepatic expression is critical in developing therapeutics targeting *APOE*. We identified several siRNA sequences that are efficacious in silencing mouse or human *APOE* and evaluated their effects *in vivo*. We show that siRNAs targeting either liver or brain *Apoe* have distinct effects on serum LDL levels. Interestingly, only complete silencing of liver *Apoe*, not partial silencing, is necessary to have detectable effects on serum LDL levels in the short-term. The impact of *Apoe* silencing on systemic cholesterol metabolism is dose-dependent, and in our hands, required almost completed (>95%) knockdown of hepatic *Apoe* mRNA and protein to have a measurable effect with the assays used.

There is a significant daily difference in circulating APOE levels, which are highly affected by diet and circadian cycles, and thus, there is a high buffering capacity within the body. Because of the buffering capacity, it appears that detectable changes in serum cholesterol require liver APOE reductions below a certain threshold. Even when liver Apoe expression was reduced by ∼75%, we found no detectable difference in cholesterol, and complete knockdown with GalNac siRNAs was needed to detect statistically significant changes in circulating cholesterol levels. Similarly, introducing Apoe at concentrations as low as 10% of normal levels has been shown to sufficiently normalize cholesterol levels in ApoE^−/−^ mice (38). These findings indicate that systemic APOE is present in significant excess and mild-to-moderate reduction of systemic APOE may not greatly impact systemic cholesterol homeostasis, at least in the short-term. However, the simple fact that we can’t detect changes does not mean there aren’t underlying physiologic effects of partially silencing liver *APOE*, and systemic APOE or cholesterol levels should be monitored in any clinical studies using *APOE* reducing agents.

Given the possible concerns of long-term, partial silencing of liver *APOE* by di-siRNAs administered in the CNS, fine-tuning of CNS versus systemic silencing is likely necessary to achieve effective and safe modulation in clinical settings. While the anti-siRNA concept is well described in the literature (20), our study shows that differentially targeting anti-siRNA and siRNAs can be used to achieve tissue-selective silencing. With these tools in hand, it is now feasible to investigate selective CNS *APOE* silencing as a potential therapeutic strategy to combat neurodegeneration.

Our anti-siRNA chemical design was largely based on results from prior studies showing 8–9 mers as the ideal length, the importance of LNA modifications within the seed, and the importance of including a cleavable linker between the conjugate and the oligonucleotide (20,37). Consistent with these reports, we observed faster and more robust efficacy with a shorter, 8-mer oligo compared with the longer 15-mer, likely due to greater affinity of the shorter sequence.

One concern with the anti-siRNA approach is the potential off target effects of the anti-siRNA, and it is possible that off target effects increase with shorter anti-siRNAs. While we designed the oligonucleotides to be complementary to the guide strand of the APOE siRNAs and to have minimal off target complements, it is possible they may bind to and block expression of miRNAs with similar sequences. In this study, the use of the GalNAc conjugate drives delivery to hepatocytes limiting the functional impact of off-target binding. However, use of non-receptor mediated conjugates and chemistries may result in greater biological significance of potential off target effects in other tissues. Evaluation of off-target effects using RNAseq will be important in future studies.

Targeting genetic causes and risk factors of disease with oligonucleotide therapeutics is an increasingly effective approach for disease-modifying therapy. Often, targets are specifically expressed in affected organs or cell types. However, like *APOE*, there are many targets with functional and important expression in non-diseased tissues. One is example is myostatin and muscle wasting. Myostatin modulation in skeletal muscles with DCA-conjugated siRNAs rescues muscle loss (39), but unwanted accumulation and silencing in cardiac muscles could result in cardiac hypertrophy (40). In addition to achieving delivery to skeletal muscles, docosanoic acid (DCA) conjugated siRNAs also deliver to and modulate myostatin expression in cardiac muscle (39), necessitating development of tools, like anti-siRNAs evaluated here, in blocking unwanted siRNA activity. In addition, siRNAs are also being investigated as potential therapeutics for cancer, including CNS cancers like glioblastoma multiforme. Potential targets for CNS tumors include Vascular Endothelial Growth Factor (VEGF), Bcl2, Stat3 and many immune related targets. To achieve therapeutic impact, high doses of tumor-targeting siRNAs into the CNS may be necessary. As many of the potential target genes are uniformly expressed across tissue and cell types, strategies to mitigate unwanted silencing in clearance and other organ systems may be necessary to reduce potential systemic side effects like VEGF-mediated hypertension or systemic immune repression. The use of anti-siRNAs may help reduce side effects that occur due to exposure of healthy tissues to therapeutic agents.

Despite the many clinical scenarios in which our approach might be useful in achieving tissue selectivity, it is important to consider its translatability. For most targets and clinical indications, tissue selective modulation is not necessary. The divalent siRNA is predominantly cleared through the liver, resulting in observable gene modulation in this tissue. Reduction in dose level supports the ability to maintain primary CNS silencing, while also reducing target gene modulation in the liver. Thus, reducing unwanted silencing in clearance tissues by dose may be a more straightforward option, but carries a risk of compromising duration of effect and necessary target reduction. As CNS administration requires direct access to CSF, which is invasive, the dosing paradigm would need to be optimized to maximize the duration and durability of silencing while reducing the frequency of administration. In such cases, administering GalNAc anti-siRNAs would provide a path for selectivity in the CNS while blocking undesired liver gene modulation. While this work provides conceptual proof of concept, using siRNAs in combination with anti-siRNAs would require more complex administration processes, more complex clinical trials, two CMCs, and two toxicity/safety studies. This situation is like that of ReversiRs: while established as a technically feasible and elegant approach to reverse siRNA activity in the liver, the clinical translatability is limited. Since its conception, the extensive preclinical optimization and overall clinical safety of GalNAc siRNAs assuaged some concerns raised that necessitated the development of tools to reverse the activity of such long-lasting compounds. Here, we show a new approach to prevent siRNA-mediated gene modulation in non-target tissues where siRNAs accumulate that may be useful in achieving safer gene modulation with new oligonucleotide chemistries. While the data presented here demonstrate proof-of-concept in the context of brain and liver APOE, this technology can be adapted to any combination of tissues using different delivery configurations and any genetic target, providing additional tools for studying and treating disease.

## Supplementary Material

gkae100_Supplemental_Files

## Data Availability

All data generated during this study are either included in this article or are available from the corresponding author on reasonable request.
