# Supplementary material for: A combinatorial approach for achieving CNS-selective RNAi
Source: Nucleic Acids Res. 2024 Feb 13;52(9):5273–84. doi: 10.1093/nar/gkae100 (PMC11109952; doi:10.1093/nar/gkae100)
Supplement: gkae100_Supplemental_Files [file gkae100_supplemental_files.zip › Supplementary Figures.pdf]

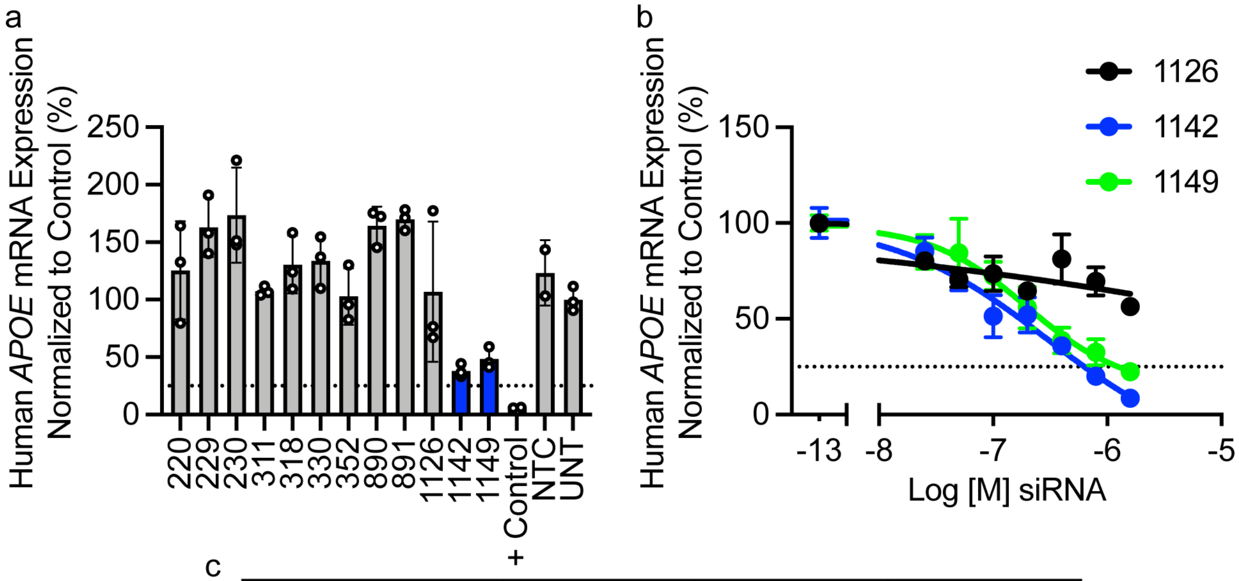

c

	APOE_1126	APOE_1142	APOE_1149
IC50 (nM)	ambiguous	298.2	19.8
Max Silencing (%)	44%	91%	78%

Supplementary Figure 1: Identification of lead siRNAs targeting human APOE. (a) Screen of 12 sequences targeting human APOE mRNA in HepG2 cells identifying 1142 and 1149 (blue) as hit sequences compared to untreated (UNT) and non-targeting (NTC) controls. A 1.5 uM siRNA dose was used to identify hit sequences. (**b**) Eight-point dose response study of two hit siRNA sequences (1142, 1149) and one less efficacious siRNA sequence (1126) in HepG2 cells confirms sequence efficacy and demonstrates low IC50 values. Dose response studies were performed by serially diluting seven times from the top dose of 1.5 uM and with the eighth point being untreated cells. (**c**) Table showing IC50 values (M) and maximum silencing (%) for siRNA hits. Cells: HepG2; Timepoint: 72 hours; APOE mRNA measured using Quantigene bDNA assay.

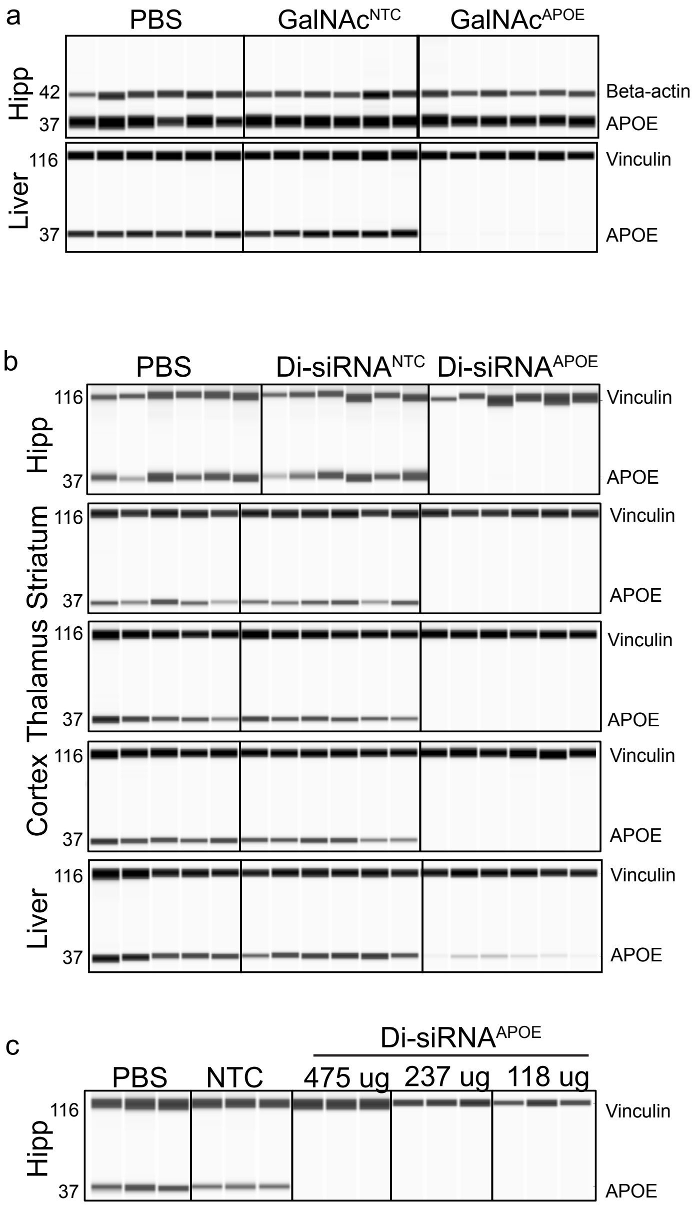

Supplementary Figure 2: Western blots showing ApoE protein silencing in wild-type mice. (a) Western blots showing ApoE silencing in the liver but not the brain after administration of GalNAc^{NTC} or GalNAc^{ApoE}. (b) Western blots showing ApoE silencing in the hippocampus, cortex, thalamus, striatum, and liver (top to bottom) after administration of di-siRNA^{NTC} or di-siRNA^{ApoE}. (c) Western blots showing ApoE protein silencing at three dose levels of di-siRNA^{ApoE} (475, 237, and 118 ug).

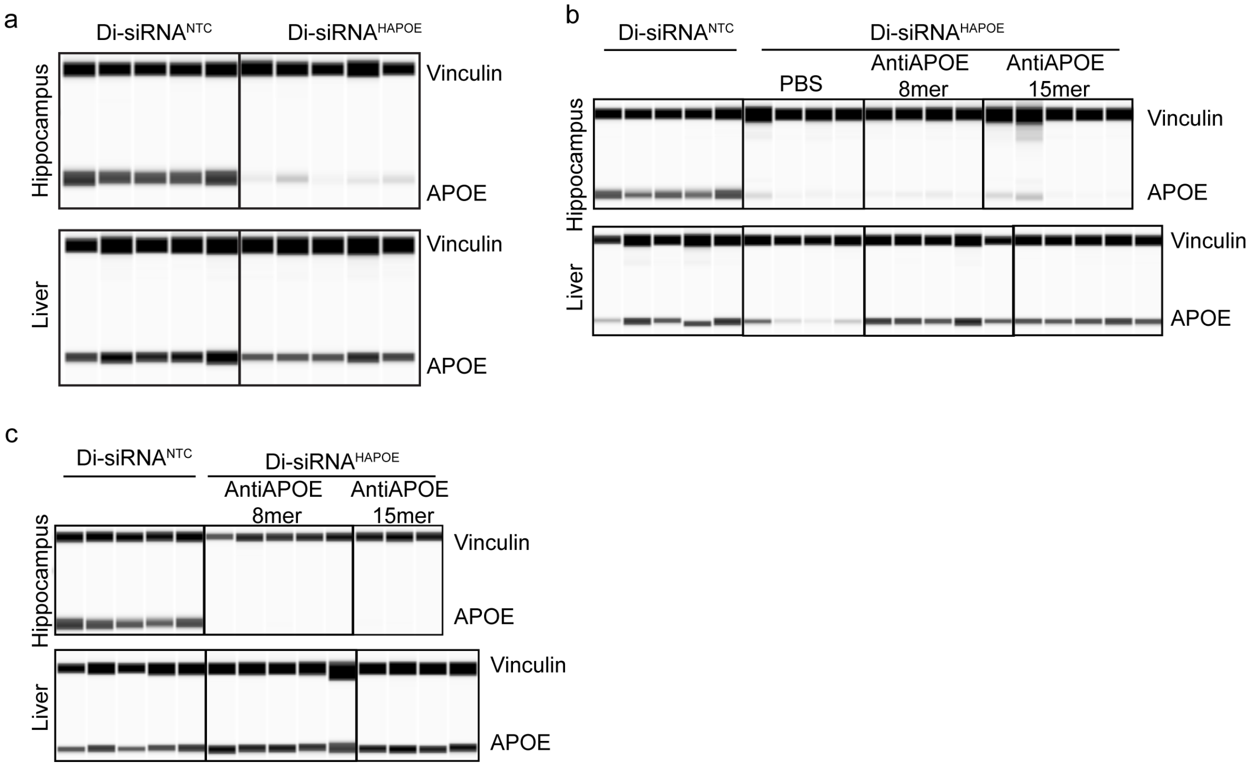

Supplementary Figure 3: Western blots showing selective modulation of CNS APOE4 in humanized APOE4 mice.
(a) Western Blots showing APOE protein (40 kDa) expression normalized to Vinculin (116 kDa) control one month post injection in the hippocampus and liver. (di-siRNA: n=5 per group, 237 µg).
(**b,c**) Western blots showing APOE protein (40kDa) expression in hippocampus and liver (normalized to Vinculin, 116 kDa) one-month after treatment with di-siRNA (475 µg, ICV) and (**b**) delayed or (**c**) concurrent administration of anti-siRNA (1mg/kg, SC; n=4-5 per group. Mice: humanized ApoE4, 8 weeks old. Tissue protein measured using the WES western blot system.
