# Supplementary material for: A combinatorial approach for achieving CNS-selective RNAi
Source: Nucleic Acids Res. 2024 Feb 13;52(9):5273–84. doi: 10.1093/nar/gkae100 (PMC11109952; doi:10.1093/nar/gkae100)
Supplement: gkae100_Supplemental_Files [file gkae100_supplemental_files.zip › Table S1_sequences.docx]

| Name | Species | Accession number | Modified Sense strand | Modified Antisense strand |
| --- | --- | --- | --- | --- |
| Di-siRNA^APOE^ | Mouse ApoE | NM_009696 | (mA)#(mA)#(fC)(mA)(fU)(mC)(fC)(mA)(fU)(mA)(mU)(mC)(fC)#(mA)#(mA)-DIO | V(mU)#(fU)#(mG)(fG)(fA)(fU)(mA)(fU)(mG)(fG)(mA)(fU)(mG)#(fU)#(mU)#(fG)#(mU)#(mU)#(mG)#(fC) |
| Di-siRNA^NTC^ | N/A | N/A | (mU)#(mG)#(fA)(mC)(fA)(mA)(fA)(mU)(fA)(mC)(mG)(mA)(fU)#(mU)#(mA)-DIO | V(mU)#(fA)#(mA)(fU)(fC)(fG)(mU)(fA)(mU)(fU)(mU)(fG)(mU)#(fC)#(mA)#(fA)#(mU)#(mC)#(mA)#(fU) |
| Di-siRNA^HAPOE^ | Human ApoE | NM_00041 | (mG)#(mA)#(fU)(mU)(fC)(mA)(fC)(mC)(fA)(mA)(mG)(mU)(fU)#(mU)#(mA)-DIO | V(mU)#(fA)#(mA)(fA)(fC)(fU)(mU)(fG)(mG)(fU)(mG)(fA)(mA)#(fU)#(mC)#(fU)#(mU)#(mU)#(mA)#(fU) |
| GalNAc^APOE^ | Mouse ApoE | NM_009696 | (mG)#(fC)#(mA)(fA)(mC)(fA)(mA)(fC)(mA)(fU)(mC)(fC)(mA)(fU)(mA)(mU)(mC)(fC)#(mA)#(mA)-GalNac | V(mU)#(fU)#(mG)(fG)(fA)(fU)(mA)(fU)(mG)(fG)(mA)(fU)(mG)(fU)(mU)(fG)(mU)(fU)(mG)(fC)#(mA)#(mG) |
| GalNAc^NTC^ | N/A | N/A | (mA)#(fU)#(mG)(fA)(mU)(fU)(mG)(fA)(mC)(fA)(mA)(fA)(mU)(fA)(mC)(mG)(mA)(fU)#(mU)#(mA)-GalNac | V(mU)#(fA)#(mA)(fU)(fC)(fG)(mU)(fA)(mU)(fU)(mU)(fG)(mU)(fC)(mA)(fA)(mU)(fC)(mA)(fU)#(mG)#(mA) |
| Anti-APOE 8mer | N/A | N/A | (mC)#(mC)#(A)#(A)#(mG)#(U)#(U)#(U)PO(A)-Galnac |  |
| Anti-APOE 15mer | N/A | N/A | (mG)#(A)#(mU)#(mU)#(mC)#(A)#(mC)#(mC)#(A)#(A)#(mG)#(U)#(U)#(U)PO(A)-GalNAc |  |

**Supplementary Table 1: Detailed sequence and chemical modification patterns of siRNAs.** Chemical modifications are designated as follows, “#” –phosphorothioate bond, “m” – 2′-O-Methyl, “f” – 2′-Fluoro, “P” – 5′ Phosphate, “V” – 5′-(*E*)-Vinylphosphonate. “DIO” – di-siRNA, “L” – Locked nucleic acid (LNA), “GalNAc” – GalNAc.
