# Supplementary material for: A combinatorial approach for achieving CNS-selective RNAi
Source: Nucleic Acids Res. 2024 Feb 13;52(9):5273–84. doi: 10.1093/nar/gkae100 (PMC11109952; doi:10.1093/nar/gkae100)
Supplement: gkae100_Supplemental_Files [file gkae100_supplemental_files.zip › Table S2_Oligo charac.docx]

| **Oligo** | **Expected mass** | **Observed Mass (g/mol)** | **Extinction Coefficient** |
| --- | --- | --- | --- |
| Mouse Di-siRNA^APOE^ Antisense | 6782.54 | 6782.6 | 200700 |
| Mouse Di-siRNA^APOE^ Sense | 10194.82 | 10194.5 | 156150 |
| Mouse GalNAc^APOE^ Antisense | 7392.64 | 7392.9 | 224910 |
| Mouse GalNAc^APOE^ Sense | 8368.27 | 8368.9 | 207180 |
| Human Di-siRNA^HAPOE^ Antisense | 6694.51 | 6696.4 | 207360 |
| Human Di-siRNA^HAPOE^ Sense | 10262.76 | 10265.1 | 153990 |
| GalNAc^NTC^ Antisense | 7264.58 | 7264.7 | 227700 |
| GalNAc^NTC^ Sense | 8451.28 | 8451.8 | 222210 |
| Di-siRNA^NTC^ Antisense | 6654.48 | 6654.7 | 203490 |
| Di-siRNA^NTC^ Sense | 10356.88 | 10359.1 | 166320 |
| Anti-APOE 8mer | 4858.35 | 4860.2 | 88380 |
| Anti-APOE 15mer | 6955.98 | 6957.3 | 150750 |

**Supplementary Table 2: Molecular weights (g/mol) and Extinction coefficients of individual oligonucleotides used in vivo.**
