# Supplementary material for: A combinatorial approach for achieving CNS-selective RNAi
Source: Nucleic Acids Res. 2024 Feb 13;52(9):5273–84. doi: 10.1093/nar/gkae100 (PMC11109952; doi:10.1093/nar/gkae100)
Supplement: gkae100_Supplemental_Files [file gkae100_supplemental_files.zip › Table S3_WT chem.docx]

|  | **2 days post injection** | | | | | | **21 days post injection** | | | | | |
| --- | --- | --- | --- | --- | --- | --- | --- | --- | --- | --- | --- | --- |
|  | **PBS (3)** | | **di-siRNA NTC (2)** | | **di-siRNA APOE (6)** | | **PBS (2)** | | **di-siRNA NTC (3)** | | **di-siRNA APOE (5)** | |
|  | Mean | SD | Mean | SD | Mean | SD | Mean | SD | Mean | SD | Mean | SD |
| ALB | 3.00 | 0.00 | 3.25 | 0.07 | 3.15 | 0.32 | 3.85 | 0.07 | 4.03 | 0.21 | 3.80 | 0.35 |
| ALP | 33.00 | 25.53 | 44.50 | 13.44 | 60.00 | 26.47 | 58.00 | 16.97 | 28.00 | 24.76 | 48.00 | 26.46 |
| ALT | 32.33 | 1.53 | 32.00 | 7.07 | 59.33 | 29.63 | 40.50 | 4.95 | 34.00 | 1.73 | 32.00 | 3.24 |
| AMY | 649.33 | 33.86 | 678.50 | 89.80 | 823.67 | 118.31 | 852.50 | 123.74 | 881.00 | 139.07 | 803.60 | 62.34 |
| Tbil | 0.15 | 0.07 | 0.15 | 0.07 | 0.18 | 0.04 | 0.25 | 0.07 | 0.20 | 0.00 | 0.25 | 0.07 |
| BUN | 24.33 | 9.07 | 23.50 | 0.71 | 24.83 | 3.87 | 25.00 | 1.41 | 19.33 | 2.89 | 19.60 | 2.30 |
| CA | 11.83 | 0.68 | 11.05 | 0.07 | 11.15 | 0.39 | 9.65 | 0.07 | 10.23 | 0.31 | 10.36 | 0.64 |
| PHOS | 7.40 | 2.43 | 8.30 | 0.42 | 6.97 | 1.11 | 7.35 | 0.78 | 6.40 | 0.69 | 7.73 | 0.83 |
| CRE | 0.25 | 0.07 | 0.25 | 0.07 | 0.28 | 0.08 | 0.20 | 0.00 | 0.20 | 0.00 | 0.20 | 0.00 |
| Glucose | 129.00 | 9.54 | 127.50 | 10.61 | 125.67 | 16.93 | 208.50 | 4.95 | 214.67 | 48.06 | 149.00 | 15.94 |
| Na+ | 154.50 | 4.95 | 155.50 | 0.71 | 138.83 | 43.06 | 145.00 | 1.41 | 142.33 | 0.58 | 145.00 | 1.00 |
| K+ | 10.25 | 2.47 |  |  | 8.58 | 0.15 | 7.35 | 0.07 | 7.40 | 0.00 | 8.50 | 0.00 |
| TP | 6.60 | 0.26 | 6.10 | 0.00 | 6.22 | 0.31 | 5.15 | 0.07 | 5.47 | 0.35 | 5.50 | 0.44 |
| GLOB | 3.60 | 0.20 | 2.85 | 0.07 | 3.05 | 0.24 | 1.30 | 0.14 | 1.43 | 0.15 | 1.37 | 0.06 |
|  | **2 days post injection** | | | | | | **21 days post injection** | | | | | |
|  | **PBS (2)** | | **GalNAc NTC (3)** | | **GalNAc APOE (4)** | | **PBS (3)** | | **GalNAc NTC (2)** | | **GalNAc APOE (5)** | |
|  | Mean | SD | Mean | SD | Mean | SD | Mean | SD | Mean | SD | Mean | SD |
| ALB | 4.00 | 0.14 | 4.00 | 0.10 | 3.90 | 0.08 | 3.87 | 0.21 | 3.70 | 0.14 | 3.80 | 0.25 |
| ALP | 101.50 | 3.54 | 98.67 | 15.28 | 132.25 | 17.63 | 53.67 | 28.73 | 65.50 | 23.33 | 74.40 | 19.24 |
| ALT | 46.50 | 0.71 | 43.67 | 4.16 | 47.50 | 7.05 | 44.67 | 2.08 | 44.50 | 4.95 | 47.60 | 11.28 |
| AMY | 893.00 | 12.73 | 975.67 | 168.30 | 891.25 | 32.84 | 868.67 | 74.57 | 799.50 | 91.22 | 909.40 | 238.66 |
| Tbil | 0.20 | 0.00 | 0.20 | 0.00 | 0.33 | 0.05 | 0.30 | 0.00 | 0.30 | 0.00 | 0.30 | 0.00 |
| BUN | 18.00 | 0.00 | 28.00 | 3.61 | 25.00 | 1.41 | 23.00 | 2.65 | 25.00 | 1.41 | 24.40 | 2.70 |
| CA | 10.50 | 0.57 | 10.20 | 0.20 | 10.13 | 0.22 | 10.13 | 0.29 | 9.90 | 0.57 | 9.68 | 0.16 |
| PHOS | 7.65 | 1.48 | 6.10 | 0.36 | 6.38 | 0.54 | 8.23 | 0.71 | 8.65 | 1.06 | 7.12 | 1.13 |
| CRE | 0.25 | 0.07 | 0.33 | 0.06 | 0.23 | 0.05 | 0.25 | 0.07 | 0.20 | 0.00 | 0.22 | 0.04 |
| Glucose | 123.50 | 17.68 | 97.00 | 43.41 | 149.25 | 7.50 | 215.67 | 40.50 | 183.00 | 15.56 | 208.00 | 52.87 |
| Na+ | 149.00 | 2.83 | 148.33 | 1.53 | 146.25 | 2.22 | 144.00 | 1.73 | 146.00 | 1.41 | 143.60 | 2.07 |
| K+ | 8.50 | 0.00 |  |  | 8.45 | 0.10 | 7.75 | 0.49 | 8.50 | 0.00 | 7.17 | 0.47 |
| TP | 5.60 | 0.28 | 5.60 | 0.10 | 5.58 | 0.15 | 5.20 | 0.36 | 5.10 | 0.00 | 5.20 | 0.14 |
| GLOB | 1.60 | 0.14 | 1.57 | 0.21 | 1.68 | 0.22 | 1.33 | 0.15 | 1.30 | 0.00 | 1.40 | 0.14 |

**Supplementary Table 3:** Blood chemistries and complete blood counts 2 days and 21 days after administration of siRNAs silencing ApoE in the brain (top) and liver (bottom).

|  |
| --- |
