# Supplementary material for: A combinatorial approach for achieving CNS-selective RNAi
Source: Nucleic Acids Res. 2024 Feb 13;52(9):5273–84. doi: 10.1093/nar/gkae100 (PMC11109952; doi:10.1093/nar/gkae100)
Supplement: gkae100_Supplemental_Files [file gkae100_supplemental_files.zip › Table S4_AntiApoE Liver markers.docx]

|  | Di-siRNA^NTC^ | | Di-siRNA^APOE^ | | Di-siRNA^APOE1156^ + PBS | | Di-siRNA^APOE^ + AntiAPOE 8mer **Reversal** | | Di-siRNA^APOE^ + AntiAPOE 15mer **Reversal** | | Di-siRNA^APOE^ + AntiAPOE 8mer  **Block** | | Di-siRNA^APOE^ + AntiAPOE 15mer **Block** | |
| --- | --- | --- | --- | --- | --- | --- | --- | --- | --- | --- | --- | --- | --- | --- |
|  | Mean | SD | Mean | SD | Mean | SD | Mean | SD | Mean | SD | Mean | SD | Mean | SD |
| ALP | 75.80 | 28.97 | 96.50 | 24.80 | 92.00 | 16.54 | 80.60 | 17.20 | 97.25 | 22.90 | 46.00 | 17.03 | 66.25 | 10.05 |
| AST | 193.40 | 94.57 | 214.50 | 103.40 | 346.80 | 194.11 | 299.20 | 105.29 | 584.00 | 412.73 | 178.20 | 22.30 | 192.00 | 71.63 |
| ALT | 42.20 | 16.10 | 35.75 | 10.78 | 69.20 | 23.08 | 65.40 | 36.71 | 235.50 | 171.22 | 27.00 | 3.32 | 36.25 | 7.37 |
| GGT | 0.00 | 0.00 | 0.00 | 0.00 | 0.20 | 0.45 | 0.20 | 0.45 | 0.25 | 0.50 | 0.00 | 0.00 | 0.00 | 0.00 |
| Albumin | 2.84 | 0.11 | 2.93 | 0.05 | 2.58 | 0.15 | 2.10 | 0.28 | 2.45 | 0.33 | 1.52 | 0.48 | 2.25 | 0.37 |
| Bilirubin | 0.02 | 0.04 | 0.00 | 0.00 | 0.00 | 0.00 | 0.00 | 0.00 | 0.00 | 0.00 | 0.00 | 0.00 | 0.00 | 0.00 |
| Glucose | 200.20 | 101.79 | 268.50 | 30.44 | 241.80 | 43.53 | 199.60 | 17.80 | 242.25 | 16.82 | 129.80 | 70.36 | 221.00 | 54.49 |
| Triglycerides | 87.60 | 15.32 | 87.75 | 17.37 | 71.80 | 4.32 | 59.20 | 13.08 | 71.25 | 19.57 | 58.20 | 20.07 | 56.75 | 4.99 |

**Supplementary Table 4:** Markers of liver toxicity in humanized ApoE4 mice after treatment with di-siRNA^APOE^ alone, di-siRNA^APOE^ + reversal with Anti-ApoE 8mer, di-siRNA^APOE^ + reversal with Anti-ApoE 15mer, di-siRNA^APOE^ + block with Anti-ApoE 8mer, and di-siRNA^APOE^ + block with Anti-ApoE 15mer. There were no detectable differences between groups.
